# Lead-vanadate sorbents for iodine trapping and their conversion into an iodoapatite-based conditioning matrix

**DOI:** 10.3389/fchem.2022.1085868

**Published:** 2022-12-21

**Authors:** R. Pénélope, L. Campayo, M. Fournier, S. Le Gallet, A. Gossard, A. Grandjean

**Affiliations:** ^1^ CEA, DES, ISEC, DE2D, Université de Montpellier, Marcoule, France; ^2^ ICB, UMR 6303 CNRS-Université Bourgogne Franche-Comté, Dijon, France; ^3^ CEA, DES, ISEC, DMRC, Université de Montpellier, Marcoule, France

**Keywords:** filter, iodine, off-gas, apatite, waste disposal

## Abstract

New lead-vanadate based sorbents were synthesized with the aim to entrap and confine gaseous iodine in off-gas streams coming from reprocessing facilities of spent nuclear fuel. Their synthesis relies on the shaping of a lead-vanadate, lead sulfide and alginic acid mix as millimetric beads. These beads were calcined between 220°C and 500°C to remove organic alginic compounds template. However, according to the calcination temperature, lead sulfide could be partially oxidized, limiting iodine loading capacity. A compromise temperature between 290°C and 350°C was found to remove most of the alginic acid template and avoiding lead sulfide oxidation. These sorbents were tested for iodine trapping in static conditions at 60°C. They performed well with a sorption capacity up to 155 mg.g^−1^ by forming PbI_2_. Furthermore, these iodine-loaded sorbents could be easily converted into an iodine-containing lead-vanadate apatite matrix by spark plasma sintering. A dense sample was produced for a sintering temperature of 500°C under 70 MPa. Such a material could be suitable for radioactive iodine conditioning in deep geological disposal. Finally, lead-vanadate sorbents could provide an easy way to entrap and confine radioactive iodine from off-gas streams into a durable material within a few steps.

## 1 Introduction

Iodine-129 generated by nuclear industry is a long-lived nuclear waste with a half-life of 15.7 million years. For spent nuclear fuels intended to be reprocessed by the PUREX (plutonium uranium extraction) process (Sakurai et al., 1992; Bertelsen et al., 2022), this isotope is essentially met in off-gas streams produced during the acidic dissolution of fuel rods. There, it has to be managed to limit its atmospheric release. Possible management strategies considered for this waste are its isotopic dilution in seawater or its trapping on solid sorbents without downstream processing. Here, we consider another possibility relying on its storage in a deep geological disposal (Von Lensa et al., 2007). Such a management solution requires iodine incorporation into an inert conditioning matrix to ensure its retention. Iodoapatite Pb_10_(VO_4_)_4.8_(PO_4_)_1.2_I_2_ is among promising matrices for this application (Audubert, 1995; Reiser et al., 2022). It can incorporate high iodine amounts (>8 wt.%) and can be consolidated into dense monoliths with densification rates higher than 95% (Yao et al., 2014; Yao et al., 2015). It also presents high chemical durability in pure water and groundwater (neutral to alkaline environments) and thus a strong iodine containment over time (Guy et al., 2002; Zhang et al., 2018; Zhang et al., 2019). The formation equation of iodoapatite from PbI_2_ can be written as follows:

$$3{Pb}_{3}{\left({VO}_{4}\right)}_{1.6}{\left({PO}_{4}\right)}_{0.4}+{PbI}_{2}\;\to \;{Pb}_{10}{\left({VO}_{4}\right)}_{4.8}{\left({PO}_{4}\right)}_{1.2}I_{2}$$ (1)

As a poorly soluble salt, PbI_2_ can be considered as an intermediate reactant to form iodoapatite. However, in nuclear fuel reprocessing plants, iodine is primarily generated in a gaseous state, mainly as I_2(g)_ (iodo-organic compounds can also be found) and, to the best of our knowledge, it is not possible to form an iodoapatite phase directly from I_2(g)_. Therefore, iodine trapping in a solid form is necessary before considering the formation of such a phase. Several solid sorbents were designed for iodine trapping such as activated carbon (Ho et al., 2019; Sun et al., 2019), graphene (Scott et al., 2015; Sun et al., 2018), Metal-Organic Frameworks (MOFs) (Xie et al., 2019; Zia et al., 2022), silver zeolites (Azambre et al., 2018; Wiechert et al., 2020), silver silica aerogel and many others. Despite the abundance of iodine sorbents (Muhire et al., 2022; Pénélope et al., 2022; Zhang et al., 2022), none has the ability to form PbI_2_ by reaction with I_2(g)_. Therefore, the synthesis of iodoapatite from existing sorbents is hardly achievable and would require either additional processing steps or, at least, a bubbling of off-gas streams into a solution containing a soluble salt of Pb^2+^. The present work tries to overcome these issues by developing sorbents that can generate PbI_2_ by reaction with I_2(g)_ and that are directly convertible into an iodine-bearing apatitic phase while minimizing the number of handling steps from the trapping of iodine to its conditioning. For that purpose, lead sulfide was chosen as a compound able to react with I_2(g)_ to form PbI_2_ on the basis of previous studies (da Silva Filho et al., 2019; Chaudhuri and Acharya, 1982) whereas, the main phase of the sorbent was selected as a lead vanadate able to react with PbI_2_ to form an iodine-bearing apatitic phase, namely Pb_3_(VO_4_)_1.6_(PO_4_)_0.4_ (Audubert, 1995).

## 2 Materials and methods

### 2.1 Synthesis of lead-vanadate sorbents

The synthesis of lead-vanadate sorbents is adapted from a previous templating procedure (Pénélope et al., 2021). The reagents used for this synthesis include alginic acid sodium salt (Sigma Aldrich), PbS (≥99.9%, Sigma Aldrich), Pb(NO_3_)_2_ (≥99.0%, Alfa Aesar) and Pb_3_(VO_4_)_1.6_(PO_4_)_0.4_ (PbVP), the latter being synthesized by thermal treatment (1,000°C for 1 h) from a mix of PbO (≥98%, VWR), V_2_O_5_ (≥99.95%, Sigma Aldrich) and NH_4_H_2_PO_4_ (≥98%, VWR) with respective molar ratios of 3.0/0.8/0.4.

**FIGURE 1:**
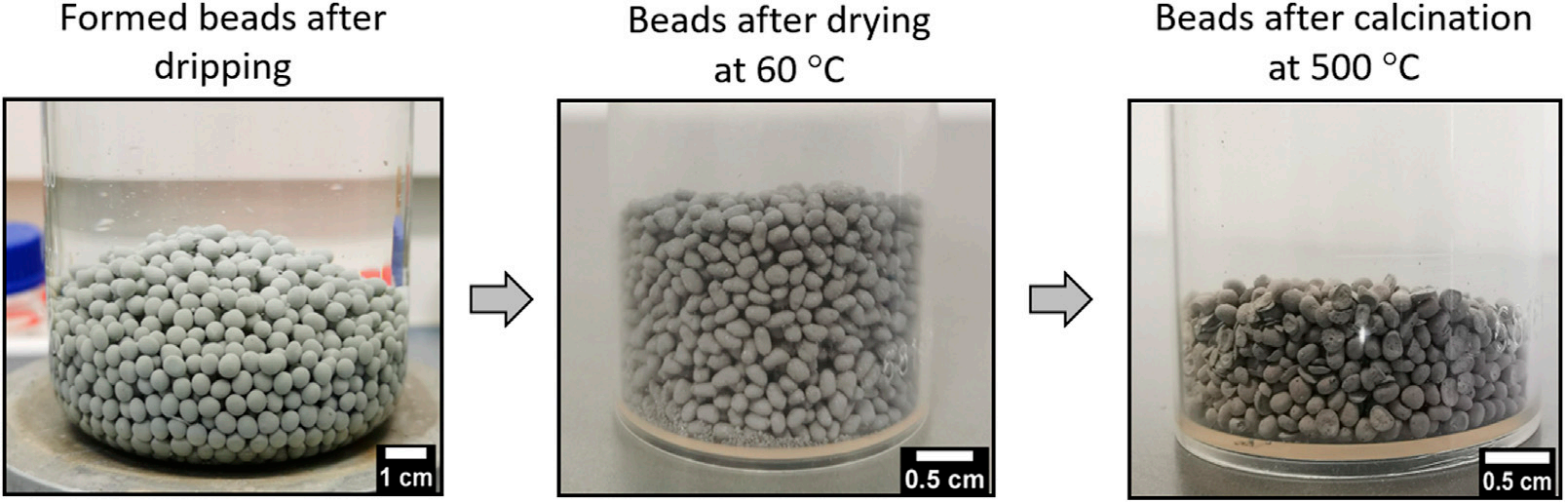
Pictures of the obtained beads calcined at 500°C at each step of the synthesis.

The first 250 ml aqueous suspension was prepared with 6 wt% of PbVP + PbS with a respective molar ratio of 2:1. Alginic acid sodium salt was then gradually added until 2 wt.% was reached and the suspension was stirred at room temperature for 4 h under magnetic stirring. A second 250 ml aqueous solution was prepared with lead nitrate at a concentration of 0.27 mol.L^−1^ and stirred for 4 h. The first suspension solution was then transferred by dripping into the second solution with a peristaltic pump REGLO Analog MS-4/8 (Ismatec^®^, Germany). A pipe of internal diameter of 2.79 mm (Cole-Parmer^®^, USA) was used for the transfer and the speed was fixed to ensure the formation of individual droplets. As a consequence, small beads are obtained in solution because of a cationic substitution between Na^+^ (from alginic acid sodium) and Pb^2+^ (from Pb(NO_3_)_2_) resulting in the reticulation of alginate network (Lee and Mooney, 2012; Abasalizadeh et al., 2020). The obtained beads were retrieved by filtration, washed twice for 1 hour in ultrapure water and once in ethanol. The beads were finally dried for 16 h in an oven at 60°C and calcined at various temperatures (220°C–500°C) under air for 90 min. The beads obtained at each of these synthesis steps are presented in Figure 1 and for a calcination temperature of 500°C.

### 2.2 Iodine capture tests in static conditions

Iodine capture tests were carried out in static conditions in a hermetic perfluoroalkoxy jar (Savillex^TM^, USA) with 4.5 g of solid iodine (I_2_, Acros Chimica) placed at the bottom. A known amount of sorbents (m_s_) (around 1.0 g) was placed above a watch glass without contact with solid I_2_. The system was hermetically closed and put in an oven at 60°C for 16 h. These conditions were chosen based on thermodynamic considerations (Riley et al., 2020). Indeed, Gibbs’ free energy of formation of iodo-complexes (for Pb^2+^) with I_2(g)_ is favorable for such temperatures compared to the formation of oxo-complexes with O_2(g)_. The jar was then cooled to room temperature for 1 h and the sorbents were weighed (m_final_) to determine the amount of trapped iodine (Δm = m_final_−m_s_). The iodine sorption capacity Q_e_ (in mg.g^‐1^) was calculated as follows:

$$Q_{e}=\frac{∆m}{m_{s}}$$ (2)

This iodine sorption capacity Q_e_ considers both chemisorbed and potentially physisorbed iodine. The physisorbed iodine amount was quantified by a gravimetric test after heating the sorbents in an oven at 150°C for 1 h. The mass loss is attributed to the physisorbed iodine amount.

### 2.3 Conversion of iodine-loaded sorbents by SPS

Iodine-loaded sorbents were converted by reactive sintering using Spark Plasma Sintering (SPS). 2.6 g of iodine-loaded sorbents were introduced into a graphite mold (15 mm inside diameter) to obtain a cylindrical matrix of 2 mm in thickness and 15 mm in diameter. Samples were pre-compacted at room temperature under 70 MPa. Then, a pressure of 40 MPa was applied for the whole heating at 500°C for 5 min (heating ramp of 50°C min^−1^). Such a temperature was chosen on the basis of the thermal stability of Pb_10_(VO_4_)_4.8_(PO_4_)_1.2_I_2_ that can range between 400°C and 500°C according to the synthesis route (Campayo et al., 2009; Le Gallet et al., 2010; Suetsugu, 2014). In the case of a reactive sintering by SPS, this thermal stability was found to be up to 500°C.

### 2.4 Characterization techniques

Field emission gun-scanning electron microscopy (FEG-SEM) was used to analyze the microstructure of the samples with a Zeiss Supra 55. Their chemical composition was also determined by energy dispersive X-ray spectroscopy (EDS) analyses with a Bruker AXS X-FlashDetector 4010 system. A probe current of 1 nA with an accelerating voltage of 15 kV at a working distance of 8.5 mm was applied. All the samples were embedded in an epoxy resin, polished (diamond paste—1 µm) and coated with a carbon layer of 10 nm. Elemental quantification with an uncertainty of 26 at% was determined from real standards spectra by using conventional PhiRhoZ correction. The collected data was processed with the ESPRIT2.0 software (Bruker™, USA).

The crystalline phases were determined by X-ray diffraction measurements (XRD) with a X’Pert PRO MPD PANalytical instrument (Bragg-Brentano geometry). A copper anode tube (Cu-Kα radiation at 0.154056 nm) working at 40 kV and 40 mA was used. Analyses were performed on powder samples at room temperature over a 2θ range of 10–90°. Diffractograms were processed with the DIFFRAC.EVA V4.2 software (Bruker™, USA) and the International Centre for Diffraction Data (ICDD) database PD4+ 2022 was used for identification purposes. Rietveld analysis was carried out with the FullProf Suite Toolbar software (January 2021 version) using the pseudo-Voigt function.

The densification rate of the obtained matrix after SPS conversion was calculated as the ratio between the hydrostatic density over the theoretical density. The theoretical density of the matrix was determined by helium pycnometry on powder samples with AccuPyc II 1340 device (Micromeritics^®^, USA). Samples were degassed under vacuum for 1 h and weighed with an uncertainty of 10^−4^ g before the measurements. The hydrostatic density was determined using Archimedes method.

## 3 Results and discussion

### 3.1 Lead-vanadate synthesized sorbents

The obtained sorbents have the same visual appearance regardless of the applied calcination temperature (220, 290, 350, 400, 450 and 500°C). An example of the obtained beads calcined at 500°C is shown in Figure 1. They are cohesive (based on a qualitative pinch test) and present a pseudo-spherical shape of 2 mm in diameter. The mass losses measured as a function of the calcination temperature are gathered in Table 1. The increase of the calcination temperature induced a higher mass loss (from 14% to 21% of loss for 220°C and 500°C respectively) mainly related to the decomposition of the alginic template. Actually, these mass variations are the result of both the decomposition of alginic acid (Eq. 3) and the potential oxidation of PbS (Eqs 4, 5) (a mass loss being indicative that the first one is the main contributor to this variation).

$${\left(C_{6}H_{8}O_{6}\right)}_{n}+5nO_{2}\;\to \;6n\left({CO}_{2}\right)+4n\left(H_{2}O\right)$$ (3)

$$PbS+2O_{2}\;\to \;{PbSO}_{4}$$ (4)

$$PbS+2O_{2}\;\to \;PbO+SO$$ (5)

**TABLE 1:** Mass losses as a function of the calcination temperature of the sorbents.

Calcination temperature (°C)	220	290	350	400	450	500
Mass loss (%)	14	18	17	18	21	21

**FIGURE 2:**
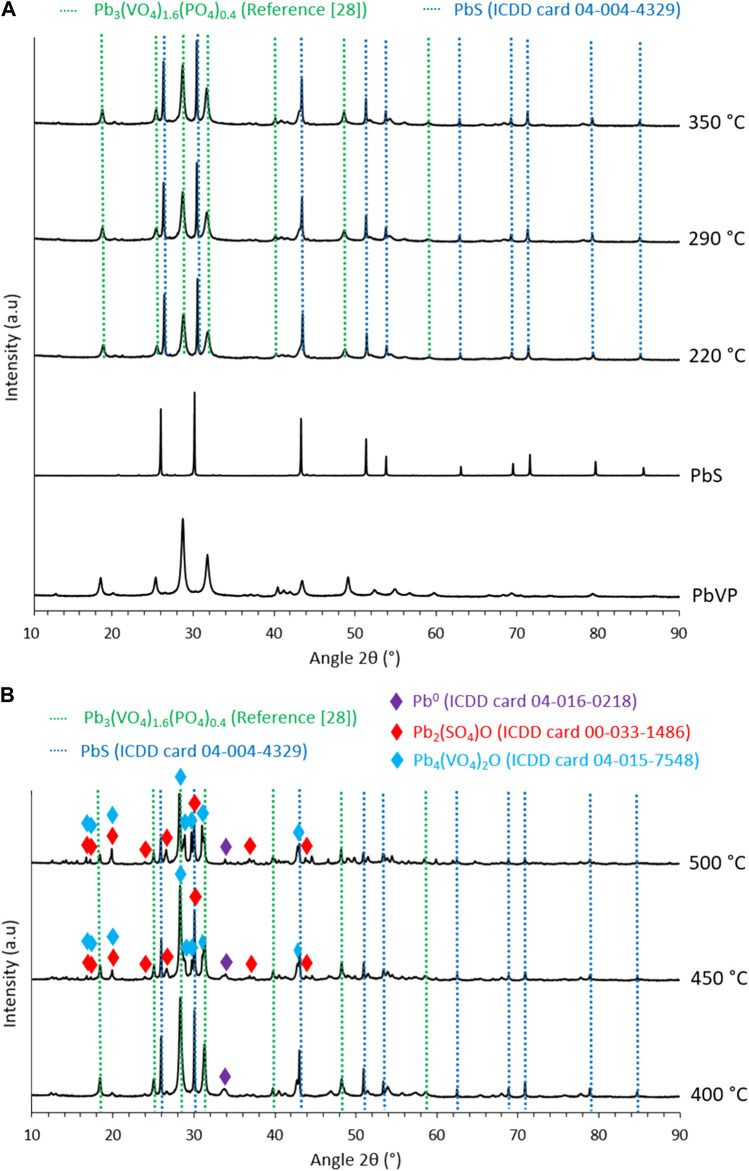
X-Ray diffractograms of **(A)** PbVP, PbS, lead-vanadate sorbents calcined at 220, 290, 350°C and **(B)** calcined at 400, 450 and 500°C.

XRD characterizations of the sorbents were carried out (Figure 2) as a function of calcination temperature to identify the potential reactions occurring upon the thermal treatment. For calcination temperatures of 220, 290 and 350°C, lead sulfide and Pb_3_(VO_4_)_1.6_(PO_4_)_0.4_ (identified by comparing the obtained diffractogram with that of Robin et al. (Robin et al., 1999)) are present. The comparison of the relative intensities of the prominent peaks of PbVP and PbS (28.4° and 30.1° respectively) revealed an increase in the intensity of the first compared to the second as the calcination temperature rose. As the formation of PbVP during calcination is excluded (no supply of vanadium and phosphorus), the reactivity of PbS was considered to be at the origin of these fluctuations. Although no additional phase could be formally identified on the diffractograms, non-indexed peaks of small intensity were observed between 40° and 42°. This indicates the formation of at least one new phase, which can be ascribed to PbS reactivity. No amorphous signal corresponding to alginate (Jana et al., 2015) was visible on the X-Ray diffractograms showing that the residual alginate content is too low to be identified by XRD. However, according to the work of Soares et al. (Soares et al., 2004), alginate residues are probably present in the calcined sorbents (from 220°C to 500°C), the complete decomposition of alginate being obtained at around 520°C.

**FIGURE 3:**
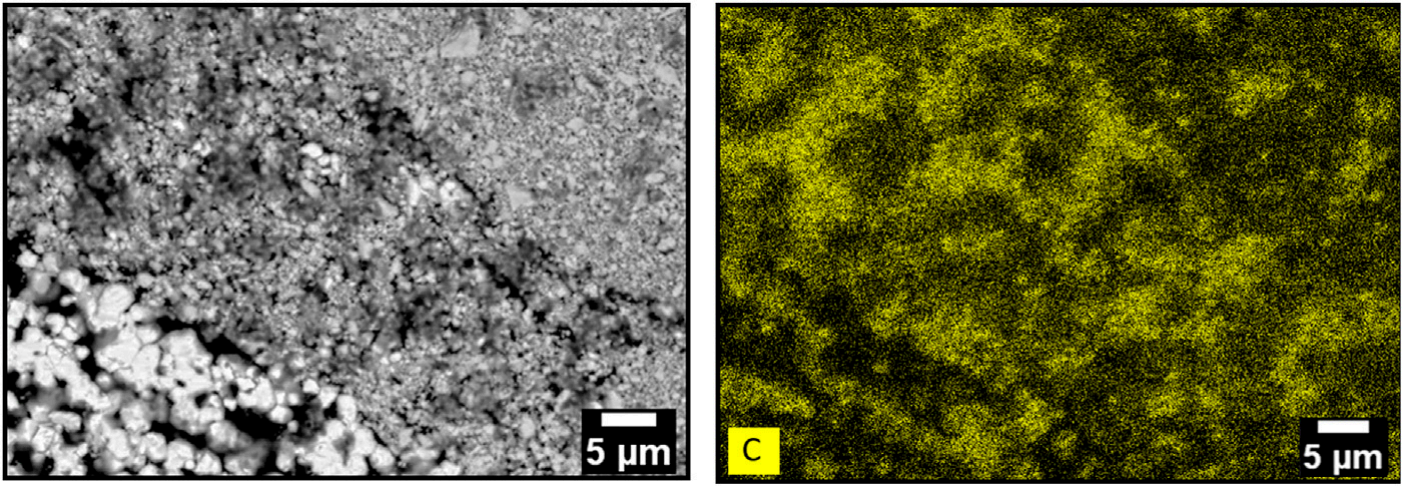
X-Ray elemental mapping analysis of a cross-section of a sorbent bead calcined at 500°C.

A similar X-ray diffractogram was observed for the sorbents calcined at 400°C even if a newly formed metallic lead (Pb^0^) was also indexed. Its presence implies a reduction phenomenon during the calcination step. Indeed, lead is initially present as Pb^2+^ form within alginate, PbVP or PbS and, alginate being mainly composed of carbon, it could impose sufficiently reducing conditions to reduce some Pb^2+^ to metallic lead. For the sorbents calcined at 450°C and 500°C, Pb_2_(SO_4_)O, a Pb_4_(VO_4_)_2_O–like phase, Pb^0^, PbVP and PbS are present. The existence of Pb_2_(SO_4_)O could result from the reaction between PbSO_4_ (generated from PbS oxidation for these calcination temperatures (Nafees et al., 2017)) and PbO coming from the oxidation of metallic lead. In the case of Pb_4_(VO_4_)_2_O (where some vanadium is substituted by phosphorus), its presence can be explained by the reactivity between PbVP and PbO, the latter one coming from the oxidation of PbS (Pb_3_(VO_4_)_1.6_(PO_4_)_0.4_ + PbO → Pb_4_(VO_4_)_1.6_(PO_4_)_0.4_O). The prominent peak of metallic lead at 34.1° is less intense when the calcination temperature increases and this could therefore align with the hypothesis suggested for Pb_2_(SO_4_)O formation. An alternative to limit this PbS oxidation would be to carry out sorbents calcination under vacuum or with N_2_ or Ar atmosphere. SEM characterizations of the sorbents calcined at 500°C (Figure 3) revealed the presence of carbonate residues derived from the decomposition of the alginic template. This confirms that, even after calcining at 500°C, not all the carbon could be removed from the beads.

### 3.2 Iodine sorption capacity of the sorbents

**FIGURE 4:**
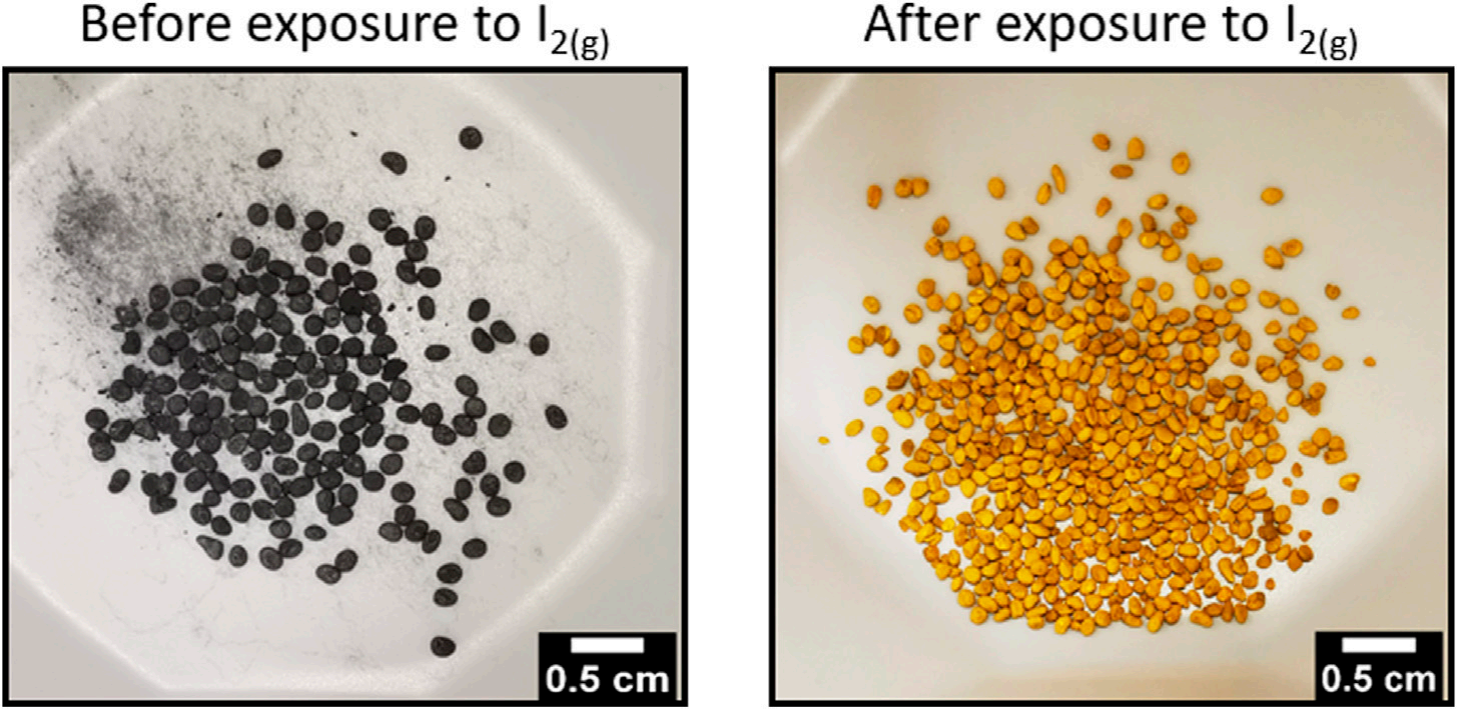
Sorbents calcined at 290°C before and after exposure to I_2(g)_.

**FIGURE 5:**
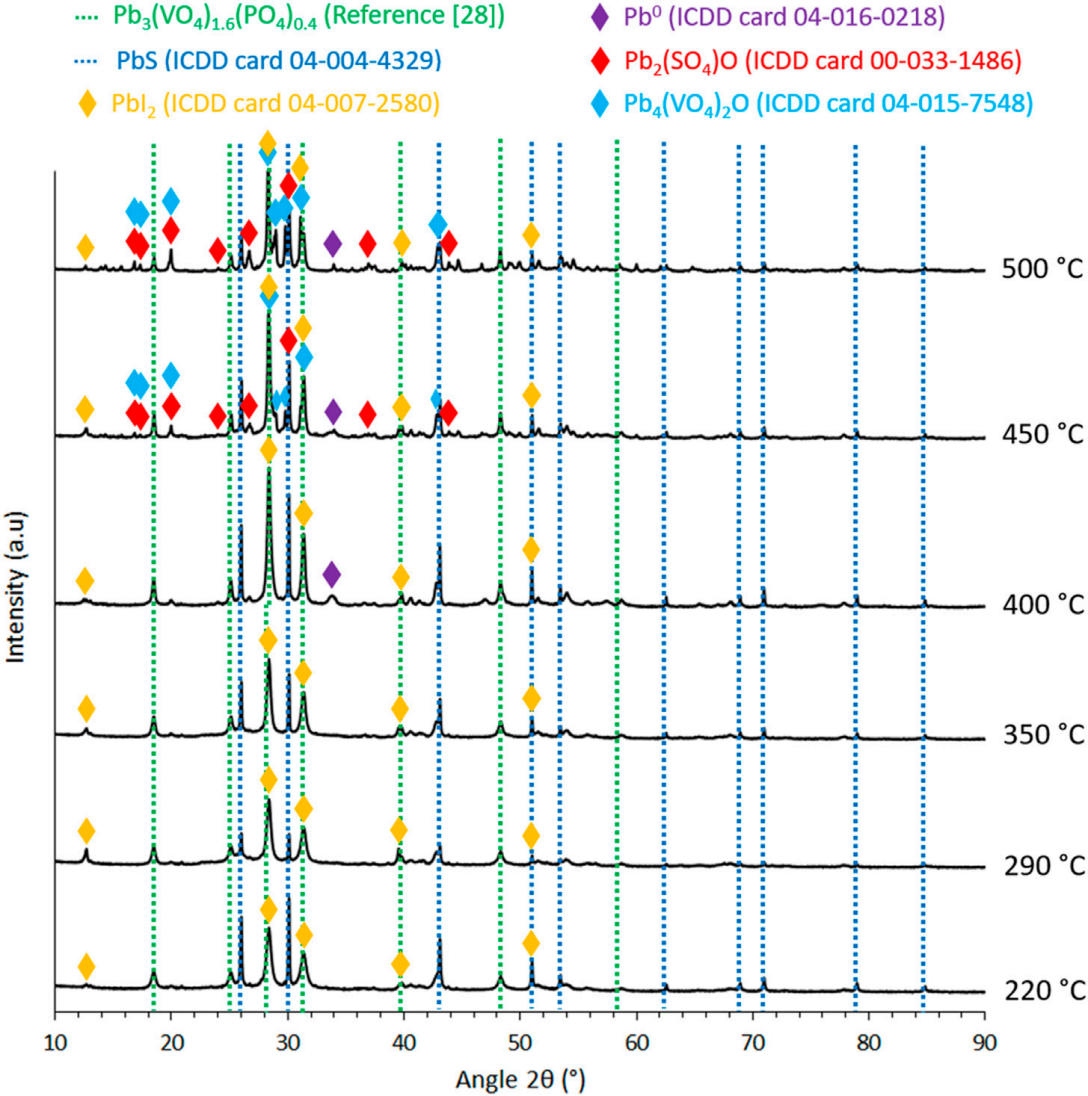
X-Ray diffractograms of lead-vanadate sorbents calcined at different temperatures after iodine capture.

As an illustration, Figure 4 shows a change in the color of the sorbents calcined at 290°C before and after exposure to I_2(g)_. This behavior was similar for all the sorbents. Their XRD characterizations after iodine exposure (Figure 5) revealed the presence of a new phase of PbI_2_. The other phases are similar to those already identified before iodine exposure (Figure 2). This demonstrates that these lead-vanadate sorbents can trap gaseous iodine by forming PbI_2_ as expected. However, the remaining presence of PbS indicates that not all the active sites react with I_2(g)_, which is probably due to their inaccessibility to iodine. The formation of PbI_2_ was accompanied by a mass gain calculated as sorption capacity using Eq. 2 and listed in Table 2.

**TABLE 2:** Iodine sorption capacity of lead-vanadate sorbents.

Calcination temperature (°C)	Not calcined	220	290	350	400	450	500
Sorption capacity (mg.g^−1^)	0	20	155	120	55	85	45

These results show a significant increase in the sorption capacity from a calcination temperature of 220°C–290°C (20–155 mg.g^−1^ respectively) and then a decrease when the temperature still increases. It is worth noting that, after a thermal treatment at 150°C for 1 h, none of these sorbents showed a significant mass loss. This reveals a negligible contribution of physisorbed iodine to the measured sorption capacities. The minimum sorption capacity was reached for the sorbents calcined at 220°C (20 mg.g^−1^). This can be explained by a significant presence of residual alginic template for such a calcination temperature, preventing access of gaseous iodine to the PbS active sites. This is consistent with the non-adsorption observed on the non-calcined sorbents due to the physical diffusion barrier constituted by alginate molecules. After calcination at 290°C, the sorption capacity reached a maximum value of 155 mg.g^−1^. In this case, the decomposition of the alginic template seems sufficiently advanced to ensure good accessibility of iodine to the PbS active sites. By increasing the calcination temperature above 290°C, the sorption capacity of the sorbents decreased from 120 mg.g^−1^ (sorbents calcined at 350°C) to 45 mg.g^−1^ (sorbents calcined at 500°C). This can be explained by the decrease in PbS content because of its oxidation at high temperature as well as by the formation of secondary phases that could prevent the accessibility of PbS particles to I_2(g)_.

### 3.3 Conversion of iodine-loaded sorbents into iodoapatite

**FIGURE 6:**
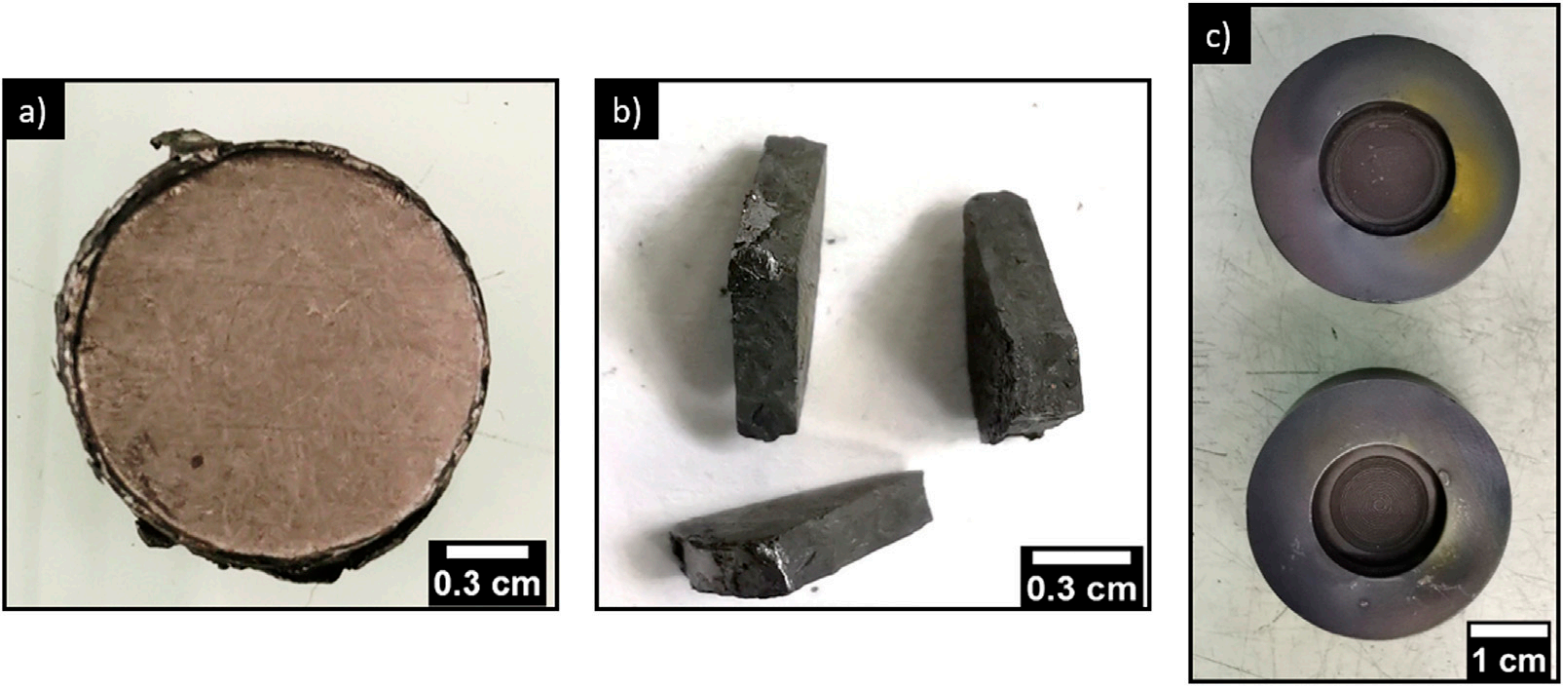
Matrix obtained after SPS conversion of iodine-loaded sorbents calcined at 350°C **(A)** in front view and **(B)** in slice view. **(C)** Graphite tools used after the sorbents conversion.

Only the iodine-loaded sorbents calcined at 350°C were converted by SPS. This sorbent was chosen because it has a high sorption capacity (120 mg.g^−1^), a satisfactory elimination rate of the alginic template and a PbVP/PbI_2_ molar ratio greater than 3 (unlike sorbents calcined at 290°C). This last characteristic is necessary to avoid over-stoichiometry of PbI_2_ compared to proportions given by Eq. 1 for iodoapatite synthesis. In this way, it limits iodine loss above 400°C, which could result from the melting of PbI_2_ if it was not entirely converted into an iodine-containing apatitic phase. The PbVP/PbI_2_ ratio was determined from the iodine sorption capacity where the content of PbI_2_ was calculated for 1 g of sorbent. The mass remaining to reach 1 g of sorbent was entirely attributed to PbVP. Therefore, the residual PbS and the carbonate residues from alginate were not taken into account, which overestimated the PbVP content and then the PbVP/PbI_2_ ratio. The obtained matrix after SPS treatment is shown in Figure 6.

Visual examination of the matrix revealed a dense material without fracture and macroscopic porosities. From its theoretical density (6.18 ± 0.01) (measured by helium pycnometry on a finely ground piece) and its hydrostatic density (6.06 ± 0.06), a densification rate of 98.1 ± 1.2% was calculated. This confirms that the matrix was dense and without significant open porosity (densification rate ≥ 92%). Note that the graphite tools, initially black, became partially yellow on the upper surface after the SPS treatment. This color change was arbitrarily attributed to a slight iodine deposit even if no characterization was performed to formally confirm this point.

**FIGURE 7:**
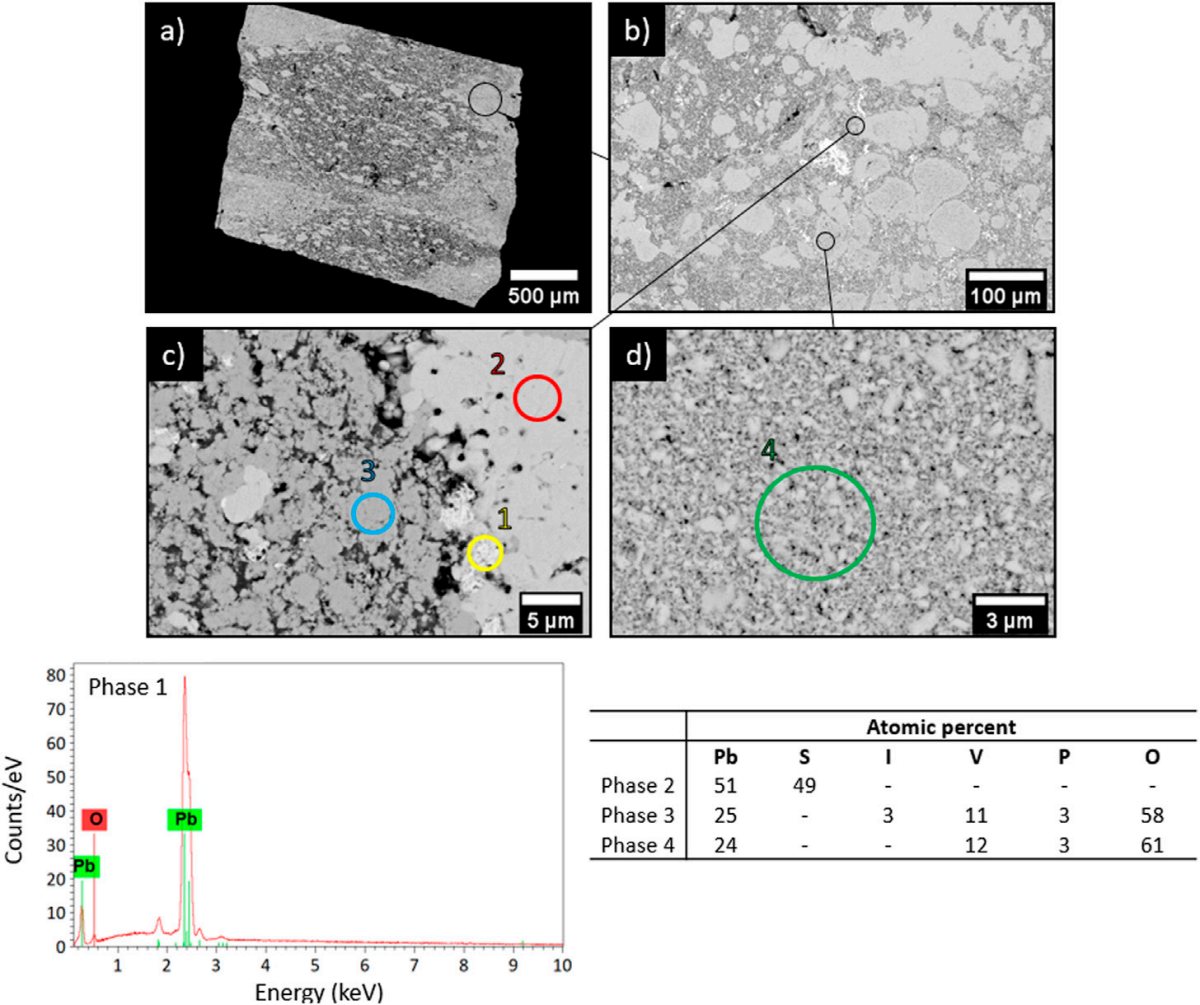
SEM pictures (BSE) **(A)** x34, **(B)** x200, **(C)** x3000 and **(D)** x6000 as well as EDS and elemental quantification of the matrix.

**FIGURE 8:**
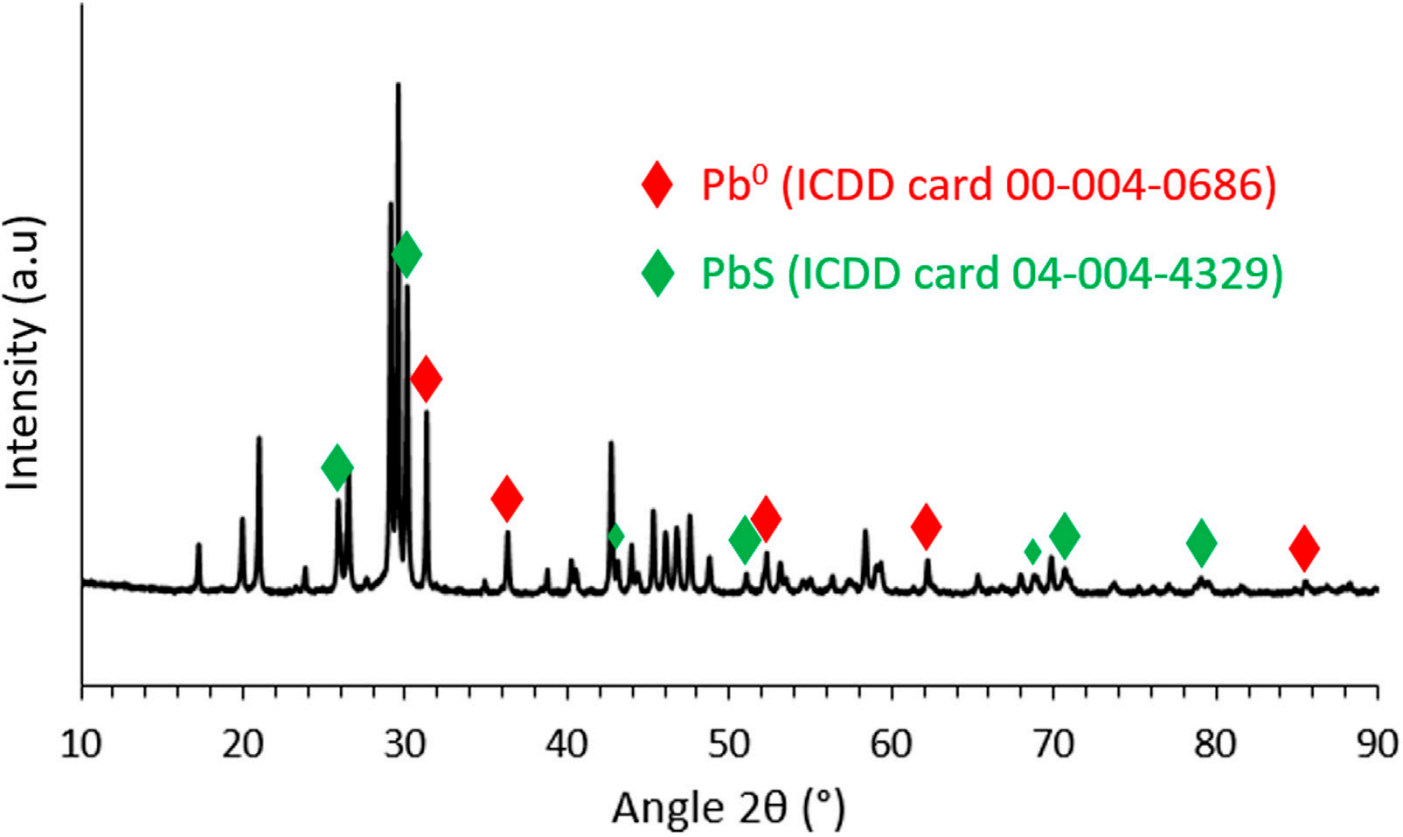
X-Ray diffractogram of the matrix.

**FIGURE 9:**
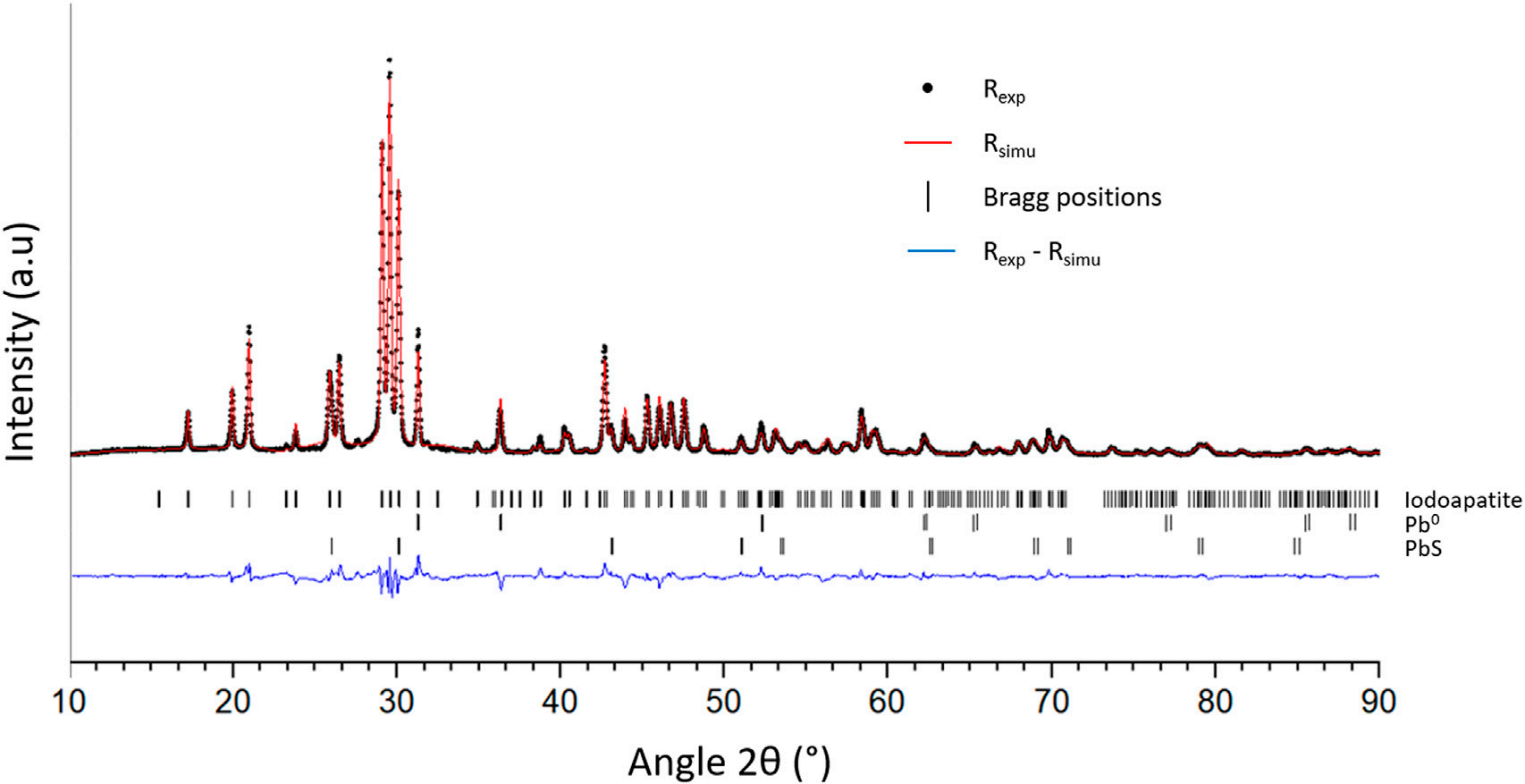
Experimental (R_exp_) and simulated (R_simu_) X-Ray diffractograms of the matrix. Bragg positions of the several phases and intensity differences between R_exp_ and R_simu_ are also presented.

SEM characterizations (Figure 7) of the obtained matrix indicate the presence of four phases. Among them, metallic lead (phase 1), PbS (phase 2) and PbVP (phase 4) are clearly identified from their chemical composition as determined by EDS. Metallic lead could come from the reduction of Pb^2+^ initially present in the calcined alginic template residues. Indeed, such a phase was also observed for sorbents treated within the same temperature range (500°C) before gaseous iodine exposure (Figure 2). For their own, PbS and PbVP phases were already present before SPS treatment and a part of them still remained after. If PbS and metallic lead can be clearly evidenced by XRD (Figure 8), this is not the case for PbVP, probably because of a low content in the sample. As well as with the absence of PbI_2_, this means that a reaction involving PbI_2_ and PbVP could happen but such a reaction did not lead to the formation of Pb_10_(VO_4_)_4.8_(PO_4_)_1.2_I_2_ as this phase was not identified. However, from elemental quantification (Figure 7), the chemical formula of the phase 3 can be written as Pb_10.7_(VO_4_)_4.7_(PO_4_)_1.3_I_1.3_O_0.9_, which is close to the composition of the targeted iodine-bearing apatite but that would exhibit a lesser iodine content. Nevertheless, assuming the presence of an iodine-bearing apatite, a Rietveld analysis of the X-Ray diffractogram was carried out to identify the crystalline phase(s) corresponding to the non-indexed peaks. The results are presented in Figure 9.

The Rietveld refinement was performed by taking into consideration Pb^0^ and PbS phases (Fm-3m space group) and assuming the presence of an iodoapatite with initial chemical formula of Pb_9.85_(VO_4_)_4.8_(PO_4_)_1.2_I_1.7_ (P6_3_/m space group). Note that site occupancies for lead and iodine were allowed to vary. This apatite composition was derived from Audubert et al. work (Audubert et al., 1999), as measured for an iodoapatite of theoretical composition Pb_9.85_(VO_4_)_6_I_1.7_ synthesized in sealed quartz tubes at 800 °C. Crystallographic data used for the refinement was retrieved from the "Crystallography Open Database” (accession numbers: 1539418 for PbS—1011119 for Pb^0^—2008226 for Pb_9.85_(VO_4_)_6_I_1.7_). By comparing the experimental X-ray diffractogram with the simulated one, a good superposition is observed and Bragg factors of 16.370, 11.390 and 9.874 were obtained for Pb^0^, PbS and iodoapatite respectively. Such results confirmed the presence of an iodoapatite phase within the matrix. The work of Audubert et al. (Audubert, 1995) demonstrated that the substitution of OH^−^ groups for I^−^ groups within an apatite structure resulted in an increase in the parameter *a* and had a negligible impact on *c*. For the iodoapatite phase present in the obtained matrix, a value of 10.283 Å for parameter *a* was calculated against 10.372 and 10.113 Å for Pb_10_(VO_4_)_4.8_(PO_4_)_1.2_I_2_ and Pb_10_(VO_4_)_4.8_(PO_4_)_1.2_(OH)_2_ respectively (Audubert, 1995). By considering a linear relation between the parameter *a* and the substitution rate of I^−^ by OH^−^, an under-stoichiometry in iodine of 33% within the obtained iodoapatite (phase 4 of Figure 7) could be calculated which agrees well with elemental composition as determined by EDS. Such an under-stoichiometry could be explained by the initial PbVP/PbI_2_ molar ratio that was slightly higher than the theoretical proportion given by Eq. 1, as well as in a slight iodine volatilization (Figure 6C). Actually, a lower thermal stability of such a kind of iodine–deficient apatite (compared to an apatitic phase having the targeted composition) could be at the origin of a slight iodine volatilization by itself. This point should have to be investigated in a future work.

## 4 Conclusion

The synthesis of a new class of lead-vanadate based sorbents able to entrap gaseous iodine and directly convertible into iodoapatite matrix by reactive sintering was demonstrated here. The synthesis of the sorbents could be achieved by an easy liquid route using an alginic organic template to form solid beads of 2 mm in diameter. Various calcination temperatures (220°C–500°C) were applied to the beads to maximize alginate removal while limiting lead sulfide oxidation which is required to entrap gaseous iodine as PbI_2_. Iodine capture tests in static conditions at 60°C indicated the highest sorption capacity of 155 and 120 mg.g^−1^ for the sorbents calcined at 290°C and 350°C. No physisorbed iodine was detected and I_2(g)_ was efficiently entrapped as PbI_2_ form. After the SPS conversion of the iodine-loaded sorbents at 500°C under 70 MPa, a conditioning matrix containing an iodoapatite phase, without residual PbI_2_, was obtained. Such a material could be suitable for radioactive iodine conditioning in deep geological disposal. Even if more studies have to be carried out (role of dynamic conditions, influence of aging in a prototypic environment …), this work hints for the first time the possibility for a sorbent to entrap gaseous iodine into PbI_2_ form and to be directly converted into a conditioning matrix by reactive sintering with a low iodine volatilization.

## Data Availability

The datasets presented in this study can be found in online repositories. The names of the repository/repositories and accession number(s) can be found below: http://www.crystallography.net/cod/- 2008226, 1011119, 1539418.
